# Genetics and breeding for climate change in Orphan crops

**DOI:** 10.1007/s00122-020-03755-1

**Published:** 2021-01-23

**Authors:** Sandra Ndagire Kamenya, Erick Owuor Mikwa, Bo Song, Damaris Achieng Odeny

**Affiliations:** 1 African Center of Excellence in Agroecology and Livelihood Systems, Uganda Martyrs University, Kampala, Uganda; 2 The International Crops Research Institute for the Semi-Arid Tropics – Eastern and Southern Africa, Nairobi, Kenya; 3 Shenzhen Branch, Guangdong Laboratory for Lingnan Modern Agriculture, Genome Analysis Laboratory of the Ministry of Agriculture, Agricultural Genomics Institute At Shenzhen, Chinese Academy of Agricultural Sciences, Shenzhen, 518060 People’s Republic of China

## Abstract

Climate change is rapidly changing how we live, what we eat and produce, the crops we breed and the target traits. Previously underutilized orphan crops that are climate resilient are receiving much attention from the crops research community, as they are often the only crops left in the field after periods of extreme weather conditions. There are several orphan crops with incredible resilience to biotic and abiotic stresses. Some are nutritious, while others provide good sources of biofuel, medicine and other industrial raw materials. Despite these benefits, orphan crops are still lacking in important genetic and genomic resources that could be used to fast track their improvement and make their production profitable. Progress has been made in generating draft genomes of at least 28 orphan crops over the last decade, thanks to the reducing cost of sequencing. The implementation of a structured breeding program that takes advantage of additional modern crop improvement tools such as genomic selection, speed breeding, genome editing, high throughput phenotyping and breeding digitization would make rapid improvement of these orphan crops possible, but would require coordinated research investment. Other production challenges such as lack of adequate germplasm conservation, poor/non-existent seed systems and agricultural extension services, as well as poor marketing channels will also need to be improved if orphan crops were to be profitable. We review the importance of breeding orphan crops under the increasing effects of climate change, highlight existing gaps that need to be addressed and share some lessons to be learned from major crops.

**Supplementary Information:**

The online version of this article (10.1007/s00122-020-03755-1) contains supplementary material, which is available to authorized users.

## Introduction

### Background

Climate change is predicted to bring about increased temperatures across the world in the range of 1.6–6 °C, and an increase in average precipitations above 2% by 2050 (Jarvis et al. 2009), triggering a host of extreme weather events including drought, flooding and heat waves (Feulner 2017). These predicted changes in climate are expected to have worldwide impacts on agriculture, with the most vulnerable areas being Africa, Asia and Latin America (Jarvis et al. 2009; Ayanlade et al. 2018). There is increasing evidence that climate change is impacting total precipitation and its temporal dynamics with significant effects on crop yields (Shortridge 2019) and biodiversity (Jarvis et al. 2009; Bálint et al. 2011; FAO 2015). One of the options for better adaptation to climate change includes the management of biodiversity for ecosystem resilience (Dauber and Miyake 2016; UNCCD 2017). A prerequisite for the use of adapted plant genetic resources in increasing the resilience of future production systems is improved knowledge of these plant resources, their origin and characterization in terms of valuable traits for climate change adaptation (FAO 2015). Unfortunately, many locally adapted varieties and plant species are orphan crops that are neglected and underutilized with a high risk of extinction before their potential roles in climate change adaptation are fully exploited (FAO 2015).

Orphan crops, which are also referred to as ‘underutilized’ (Dawson and Jaenicke 2006), ‘minor’ (Umesh et al. 2019), ‘neglected’ (Hendre et al. 2019; Tadele 2019; Popoola et al. 2019), ‘promising’ (for emerging markets, or because of previously unrecognized valuable traits), ‘niche’ (of marginal importance in production systems and economies) and/or ‘traditional’ (used for centuries or even millennia) crops (Gregory et al. 2019), are crops with important attributes, not globally known, have the potential to be grown for profit or subsistence, have been under-researched in the past and, therefore, have inadequate or total lack of genetic and genomic resources. Despite their neglect in research and investment, orphan crops have the potential to address multiple UN Sustainable Development Goals in the low-income nations of Africa (Hendre et al. 2019), Asia (Gregory et al. 2019) and Latin America, as well as in the growing western consumers’ interests in new healthier foods (Dawson et al. 2019).

Most orphan crops are generally more adapted to the extreme soil and climatic conditions as they contain the relevant alleles and mechanisms for growth in poor environments and for resilience under stress (Oibiokpa et al. 2014; Tátrai et al. 2016) that have potentially been lost from major crops (Ellstrand et al. 2010; Cullis and Kunert 2017). Orphan crops have been recognized as potential sources of resilience traits (Chiurugwi et al. 2019) that can be used to improve major crops and also play a role in improving sustainability of food systems (Mabhaudhi et al. 2019; Borelli et al. 2020; Dawson et al. 2019). This increasing recognition of the important role of orphan crops has resulted in the launch of advanced research and development initiatives (Tadele and Bartels 2019). Other traits of importance include nutrition (Dawson et al. 2019), medicinal value (Tlili et al. 2011), biofuel (King et al. 2015), cosmetics (Saikia and Konwar 2012) and for feed/fodder (Tolera and Sundstøl 2000). A summary of select orphan crops and their importance is presented in Supplementary Table 1. We further discuss the key contributions of orphan crops globally under four sub-topics below.

### Resilience to biotic stresses

A recent study estimated significant economic losses in major crops as a result of pests and diseases and recommended the prioritization of plant health to improve the sustainability of agro-ecosystems (Savary et al. 2019). Part of the solution lies in the diversification of agro-ecosystems using orphan crops, majority of which have been reported to show tolerance to some of the pests and diseases (Hendre et al. 2019). Forage legumes in the genus *Desmodium,* mainly *D. uncinatum* and *D. tortum*, have been used to suppress one of the most devastating parasitic weeds in Africa, *Striga hermonthica* (Midega et al. 2017)*. Striga* is a parasitic weed to most cereals including maize (*Zea mays* L.), sorghum [*Sorghum bicolor* (L.) Moench] and rice (*Oryza sativa* L.). *Desmodium* spp., an orphan crop, suppresses *Striga* when intercropped with cereals (Midega et al. 2010) through a combination of different mechanisms including the production of an allelochemical that inhibits the radicle growth of *Striga* (Hooper et al. 2010). Spider plant (*Gynandropsis gynandra* (L.) Briq.) has been reported to significantly reduce the incidence of thrip species *Megalurothrips* and *Frankliniella occidentalis* (Waiganjo et al. 2007) when used as a companion crop with snap bean (*Phaseolus vulgaris*), while finger millet (*Eleusine coracana* subsp. *coracana*) is effective in suppressing weed growth (Samarajeewa et al. 2006).

Other orphan crops have been used as donors of resistance genes that were successfully introgressed into major crops. Within the Solanaceae family, the African eggplant (*Solanum aethiopicum*), which is used as a vegetable in Africa, is a source of resistance to *Fusarium oxysporium* f. sp. *melongenae* (Rizza et al. 2002; Toppino et al. 2008) and *Ralstonia solanacearum* (Collonnier et al. 2001) for the improvement of other Solanaceae crops. *S. aethiopicum* rootstocks have been reported to improve disease resistance in tomato (*Solanum lycopersium* L.) (Nkansah et al. 2013), while *Solanum torvum,* also an orphan vegetable, is the preferred rootstock for improved resistance to diseases in brinjal eggplant (*Solanum melongena*) (Sakata et al. 1989; Ramesh et al. 2016). Watermelon (*Cucumis melo*) grafted onto the rootstock of the little known bottle gourd (*Lagenaria siceraria*), conferred resistance to *Fusarium* spp. (Davis et al. 2008).

### Resilience to abiotic stresses

Many orphan crops have been identified as climate-smart and able to adapt to the ever-changing climate of their respective agro-ecological regions. Drought is one of the major abiotic constraints limiting agricultural production worldwide alongside low temperatures, soil salinity, nutrient deficiencies and toxic metals (Shinozaki et al. 2015). There are several orphan crops that have been reported to exhibit high levels of tolerance to drought stress, although the stability of their yields, in most cases, has not been established. Such crops include finger millet (Neshamba 2010; Krishnamurthy et al. 2016), foxtail millet (*Setaria italica*) (Puranik et al. 2011), fonio (*Digitaria* spp.) (Vietnameyer et al. 1996), grass pea (*Lathyrus sativa* L.) (Hanbury et al. 2000) and quinoa (*Chenopodium quinoa* Willd.) (Hinojosa et al. 2018). The drought mechanisms reported in these orphan crops include efficient antioxidant potential (Puranik et al. 2011; Bhatt et al. 2012; Jiang et al. 2013), association with arbuscular mycorrhiza (Tyagi et al. 2017), osmotic adjustment (Jiang et al. 2013; Tyagi et al. 2017), reduction in the green leaf area and stomatal conductance (Cullis and Kunert 2017). Caper (*Capparis spinosa* L.), an orphan shrub cultivated for its flower buds and fruits in the Mediterranean, shows remarkable resilience to heat stress (Levizou et al. 2004).

Other orphan crops have developed genetic and molecular mechanisms to survive in poor soils of low fertility where most of the major plants do not grow (Naluwairo 2011; Takada et al. 2017; Cullis et al. 2018; Mabhaudhi et al. 2019). Finger millet grows successfully on marginal lands with poor soil fertility (Thilakarathna and Raizada 2015) and exhibits a higher degree of salt tolerance in comparison to other cereals (Bray et al. 2000; Shailaja and Thirumeni 2007; Rahman et al. 2014). Common glasswort (*Salicornia europaea* L.), an orphan annual dicot with diverse uses (Loconsole et al. 2019), is one of the most salt-tolerant species worldwide (Patel 2016). Other orphan crops have been used in phytoremediation (Mkumbo et al. 2012), the most sustainable way of rehabilitating polluted lands through the use of plants to extract heavy metals from soil (Raskin et al. 1997). Plants of the Amaranthaceae family, including *Salicornia brachiata* (Sharma et al. 2010), *Amaranthus spinosus* (Chinmayee et al. 2012), *Amaranthus retroflexus* L. var. retroflexus and *Amaranthus hybridus* L., have been reported to be tolerant to various heavy metals (Mohsenzade et al. 2009; Zhang et al. 2010). African yam bean (AYB) (*Sphenostylis stenocarpa* Harms) and *Jatropha curcas* have been reported as excellent phytoremediators for heavy metal (i.e., Al, Fe, Cr, Mn, Ar, Zn, Cd and Pb) contaminated soil (Jamil et al. 2009; Ochekwu and Eneh 2012; Chandra et al. 2016a). These examples present opportunities for the promotion of these crops to a higher level of production.

### Medicinal/pharmaceutical/cosmetic value

Many people in the developing countries have depended on orphan crops for medicine, pharmaceuticals and cosmetics for centuries. The African eggplant has been used traditionally in the management of a range of ailments from weight reduction and hypertension (Miller et al. 1999; Odetola et al. 2004; Ogunka-Nnoka et al. 2018) to treatment of several conditions such as diabetes (Ezugwu et al. 2005), anticonvulsant (Gbile and Adesina 1988), skin infections (Oliver-Bever 1986), rheumatic disease, swollen joint pains (Anosike et al. 2012), colic, ulcers, gastro-esophageal reflux disease and constipation (Gbile and Adesina 1988; Ezugwu et al. 2005). In addition to the high levels of vitamin C and β-carotene, the African eggplant has significant levels of alkaloids, saponins, flavonoids, tannins, ascorbic acid and steroids (Chinedu et al. 2011a, b; Neugart et al. 2017; Sekulya et al. 2018) making it a potential source of precursors for pharmaceutical drugs. There are several reports on the medicinal properties of *Solanum anguivi* (Ripperger and Himmelreich 1994; Elekofehinti et al. 2012, 2013, 2015), confirming its traditional use as medicine in certain parts of Africa. Breadfruit {*Artocarpus altilis* (Parkinson) Fosberg} contains fatty acids and extracts that are used in pest management (Jones et al. 2012; Eccles et al. 2019) and has traditionally been used in Asia for the treatment of malaria, yellow fever, dengue fever (Jacob et al. 2015), liver cirrhosis, hypertension and diabetes (Wang and Wang 2010; Jones et al. 2012).

Finger millet grain, which is gluten free, has been used in the management of physiological disorders such as diabetes, hypertension, vascular fragility, hypercholesterolemia, prevention of oxidation of low-density lipoproteins (LDLs) and also to improve gastrointestinal health (Chandra et al. 2016a, b; Kumar et al. 2016; Chethan and Malleshi 2007). Tef (*Eragrostis tef*) has similarly received attention as a life-style crop due to its gluten-free nature (Spaenij-Dekking et al. 2005; Tadele 2019). Regular consumption of the spider plant (*Cleome gynandra* L.) by expectant mothers has been reported to relieve childbirth complications as well as reduce the length of the labor period (Onyango et al. 2013). Different parts of the spider plant have been used as a relief for epileptic fits, ear, eye and nostril aches, for the treatment of inflammations, headaches, scurvy, marasmus (Opole et al. 1995; Narendhirakannan et al. 2005), neuralgia, rheumatism (Chweya and Mnzava 1997) and diabetes (Shaik et al. 2013). Shea butter extracted from the shea tree (*Vitellaria paradoxa* or *V. nilotica*) is often used as a base in medicinal ointments due to its anti-inflammatory properties (Maanikuu and Peker 2017).

The cosmetic industry has benefited from the shea tree products as one of the best anti-ageing and moisturizing agents for the skin with sun-screening and collagen boosting  properties (Suter et al. 2016; Montenegro and Santagati 2019). The African melon (*Acanthosicyos horridus* and *Citrullus lanatus*) has been reported to have great potential in both the food and cosmetic industry (Houdegbe et al. 2016; Cheikhyoussef et al. 2017), while the emulsifying capacity of biosurfactants from quinoa has been reported as suitable for incorporation into cosmetic emulsion formulations (Bezerraa et al. 2020). The seeds of marama bean (*Tylosema esculentum*) have traditionally been consumed and used as a cosmetic by the natives of the Kalahari (Cullis et al. 2019), while tiger nut (*Cyperus esculentus*) oil is commonly used as a cooking ingredient and in skin care (Ezeh et al. 2014).

### Sources of other novel traits (nutrition, feed/fodder, biofuel)

Orphan crops play an important role in the economies of many countries, particularly in the developing world, as sources of nutrition (Jamnadass et al. 2020). It is believed that breeding for increased production of orphan crops can reduce malnutrition and stunting (Bekkering and Tian 2019; Tadele 2019). *Amaranthus hypochondriacus* leaves, for example, contain far more vitamin A as compared to other green leafy vegetables like spinach and cabbage (Hunter et al. 2019). Varieties of finger millet are way more nutritious than white rice in their calcium, iron, potassium, magnesium and zinc content (Tripathi and Platel 2010). The high levels of minerals, vitamins and fats in yams (*Dioscorea* spp.) outperform the commonly consumed potatoes (Padhan and Panda 2018, 2020). These exceptional nutritive qualities have been a major target for improving resource availability of orphan crops through whole genome sequencing (Jamnadass et al. 2020).

Several legume orphan crops species are cultivated for food, feed and fodder including winged bean (*Psophocarpus tetragonolobus* L.) (Hyland 1968; Hymowitz and Boyd 1977), hyacinth bean (*Lablab purpureus* L.) (Morris 2009a, b), lima bean (*Phaseolus lunatus* L.) (Andueza-noh et al. 2015), jack and sword bean (*Canavalia* sp.) (Akpapunam and Sefa-Dedeh 1997), mung bean (*Vigna radiata* L.) (Tang et al. 2014), bambara groundnut (*Vigna subterranea* L.) (Mayes et al. 2019), marama bean (*Tylosema esculentum* L.) (Cullis et al. 2019), kersting’s groundnut (*Kerstingiella geocarpa* Harms) (Ayenan and Ezin 2016), African yam bean (AYB) (Asare et al. 1984) and rice bean (*Vigna angularis* L.) (Joshi et al. 2008). All parts of the enset plant (*Ensete ventricosum*) are used for animal forage (Borrell et al. 2019).

The rising demand for biofuels has led to the identification of orphan crops as sources of ‘second-generation’ biofuels. The seeds of an orphan crop, *Jatropha curcas* (Physic nut or Jatropha), that contain 27–40% oil can be processed to produce a high-quality biodiesel fuel, usable in a standard diesel engine or further processed into jet fuel (Duraes et al. 2011; Odeh and Tan 2015). The residue (press cake) is used as feed in digesters and gasifiers to produce biogas, or as biomass feedstock to power electricity plants, or as a fertilizer due to its nitrogen, phosphorus and potassium content (Achten et al. 2007). The seeds of the African melon (*Cucumeropsis mannii*) have also been shown to have applications in biodiesel production (Dansi et al. 2012; Houdegbe et al. 2016).

## Genetics and breeding of select climate smart orphan crops

**Fig. 1:**
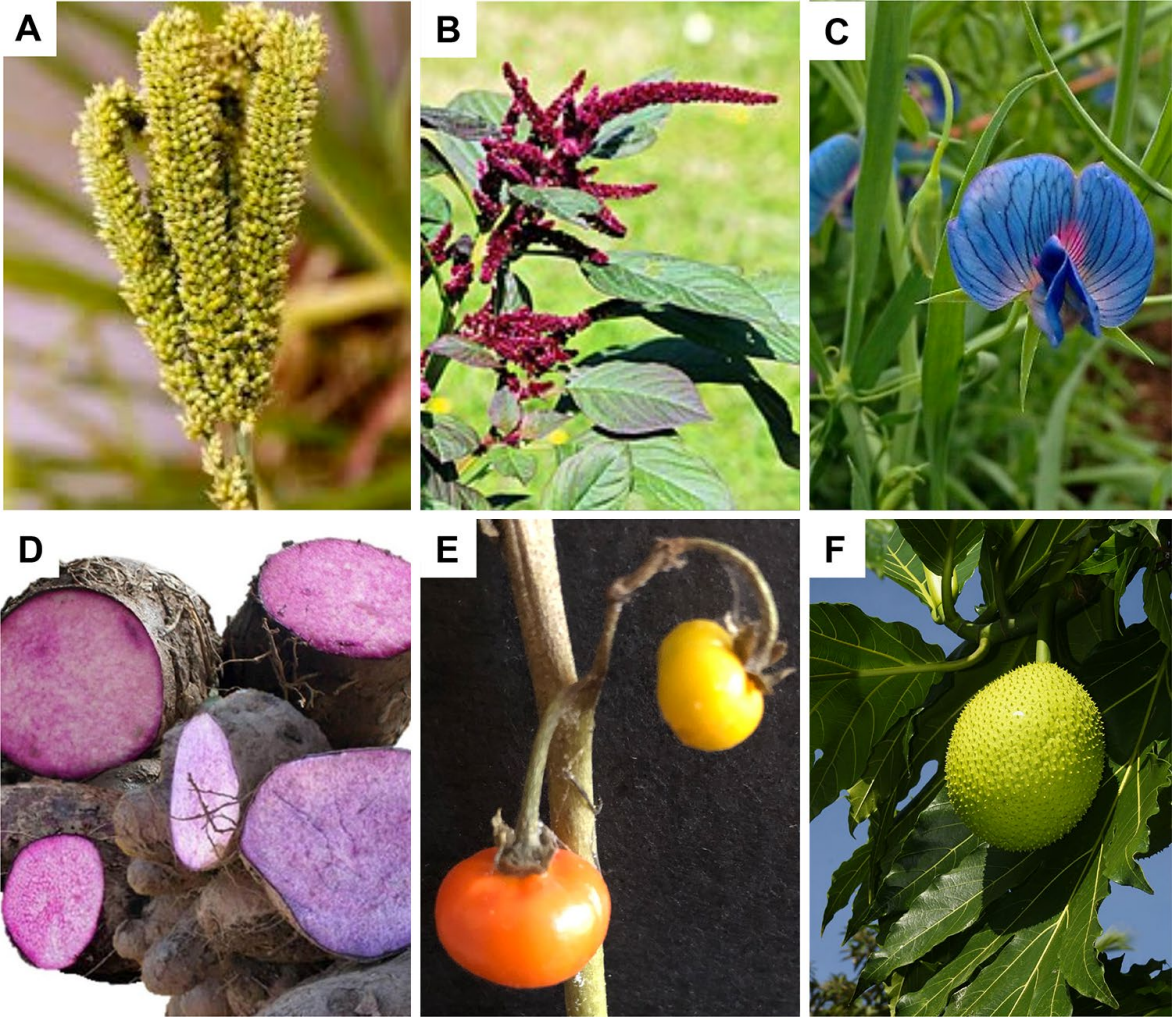
Pictures of orphan crops finger millet (**A**), grain amaranth (**B**), grass pea (**C**), water yam (**D**), African eggplant (**E**) and breadfruit (**F**)

Despite their demonstrated economic importance and their beneficial contributions to agro-ecosystems, there has been a lag in the overall genetic improvement of orphan crops. The breeding methods are conventional, slow and lacking in innovation, while the breeding objectives are not well defined beyond the enhancement of domestication syndrome traits. In this section, we discuss the genetics and breeding of six climate-resilient orphan crops (Fig. 1), randomly selected from the main categories of food crops, cereals, pseudo-cereals, legumes, root and tuber crops, vegetables and fruits. These crops are seen as most promising climate resilient crops for the future and are already receiving global attention, including being incorporated as mandate crops of some of the Consultative Group of International Agricultural Research (CGIAR) centers, in some cases. For each of the crops, we briefly discuss their origin, domestication, distribution, genetic resources, economic importance and breeding status.

### Finger millet

Finger millet (*Eleusine coracana* subsp. *coracana*) (2*n* = 4*x* = 36) (AABB) is an annual orphan cereal crop belonging to Poaceae family and Chloridoideae sub-family (Srinivasachary et al. 2007). There are seven other species of annual grasses in the genus *Eleusine*, including *E. kigeziensis, E. indica, E. intermedia, E. floccifolia, E. tristachya, E. jaegeri and E. multiflora*. Finger millet is believed to have been domesticated from its wild progenitor, *E. coracana* subsp. *africana,* about 5000 years ago (Dida et al. 2007). It is the only cultivated crop of the genus *Eleusine* and has four cultivated races, namely, *elongata, plana, compacta* and *vulgaris* (Upadhyaya et al. 2010). *E. indica* (AA) is the AA genome donor, while the BB genome donor remains unknown. More than 37,000 wild and cultivated finger millet germplasm has been conserved globally (Vetriventhan et al. 2016) in various gene banks, with the National Bureau of Plant Genetic Resources in India having the highest number of collections (> 10,000) followed by the International Crops research Institute for the Semi-Arid Tropics (ICRISAT) (7519) (Odeny et al. 2020). East Africa and India are considered the primary and secondary centers of diversity, respectively (Bisht and Mukai 2002) for finger millet, and accessions from the two regions appear to be genetically and morphologically distinct (Arya et al. 2013; Babu et al. 2014a; Ramakrishnan et al. 2015; Puranik et al. 2020).

Finger millet is largely cultivated for its nutritious gluten-free healthy grain and resilience to several biotic and abiotic stresses (Rodriguez et al. 2020). Traditionally, genetic improvement in finger millet was limited to pedigree-based selection for larger seed size, higher yield and less shattering, with a focus on enhancing its domestication. The inclusion of finger millet as a mandate crop of ICRISAT led to a relatively more structured breeding with the main objectives as enhancing resistance to blast disease (*Magnaporthe grisea* teleomorph: *Pyricularia grisea*), *S. hermonthica* (parasitic witchweed), lodging, tolerance to stressful soil and moisture conditions, and grain that can be more easily dehulled and ground (National Research Council 1996). Hybridization in finger millet has been undertaken manually, or using the plastic bag technique, hot water treatment, or the use of chemicals (Kunguni et al. 2015) due to the lack of cytoplasmic male sterile (CMS) lines. Its self-pollinating nature (Hilu and de Wet 1980) and the tiny floral architecture have hindered bulk hybridizations, especially in Africa, where most improved varieties released are from selections. Most of the hybridization-based breeding has been done in Asia and, in some cases, included African germplasm leading to the release of ‘*Indaf*’ varieties (Deba et al. 2008) with improved yields. There are now a few programs in E. Africa employing hybridization-based breeding through hand emasculation and pollination.

Genomics-assisted breeding has been limited in finger millet due to lack of a robust set of molecular markers until recently. The first partial finger millet genetic map was constructed by Dida et al. (2007) using Restriction Fragment Length polymorphism (RFLP), Amplified Fragment Length Polymorphism (AFLP) and Single Strand Conformation Polymorphic (SSCP) expressed sequenced tags. The map was constructed using an interspecific F_2_ mapping population between *Eleusine coracana* subsp. *coracana* (Okhale 1) and *Eleusine coracana* subsp. *africana* (MD-20), and contained 327 loci that were mapped to either A or B genomes. More recently, a more robust single nucleotide polymorphism (SNP) linkage map was developed using F_2:3_ families of the same interspecific cross between Okhale 1 and MD-20 (Qi et al. 2018). This recent map used 4453 SNP markers in 18 linkage groups that were designated the same as in Dida et al. (2007) and incorporated a subset of markers that had been mapped in the first linkage map. There is currently no linkage map generated exclusively from a cross involving the cultivated subspecies only. Trait mapping has been limited in finger millet, and the few studies undertaken so far used association mapping, albeit with less than optimal numbers of genotypes. Most of the traits mapped are agronomic (Babu et al. 2014a; Lule et al. 2018; Sharma et al. 2018), although there are also reports of association mapping for blast disease resistance (Babu et al. 2014b) as well as for nutrition-related traits (Babu et al. 2014c; Puranik et al. 2020).

### Amaranthus hypochondriacus

*Amaranthus hypochondriacus* (2*n* = 2*x* = 32) is one of the more than 70 species of the genus *Amaranthus*, mostly annuals, distributed across the world’s tropical and temperate regions. It is also one out of the three *Amaranth* grain species considered to have desirable agronomic characteristics besides *A. cruentus* L. and *A. caudatum* L (Grubben and Denton 2004) that form part of the hybridus complex together with the two potential wild ancestors, *A. hybridus* and *A. quitensis*. *A. hypochondriacus* crosses easily with the four species within the hybridus complex making it difficult to fully understand the taxonomic relationships within the complex. There are several hypotheses on the origin of the three-grain amaranths (Sauer 1967,1976; Kirkpatrick 1995; Xu and Sun 2001; Mallory et al. 2008) although more recent molecular evidence supports a monophyletic origin (Kietlinski et al. 2014; Viljoen et al. 2018), perhaps from two or three different, geographically separated lineages of *A. hybridus* (Stetter and Schmid 2017; Stetter et al. 2020). The first record of *A. hypochondriacus* domestication was by the Aztec civilization in central Mexico (Brenner et al. 2010) but most of its cultivation today occurs in India. Approximately 61 diverse collections of amaranth genetic resources are being maintained in at least 11 countries of the world (Das 2016; Joshi et al. 2018), majority of which reside in the USDA germplasm collection with a total of 3338 accessions from 40 countries (Trucco and Tranel 2011; Brenner 2015).

*Amaranthus hypochondriacus* is cultivated mainly as a pseudo-cereal but also for fodder (Abbasi et al. 2012) and as an ornamental (Pandey and Singh 2011). The grains are highly nutritious (Ssepuuya et al. 2018) with medicinal properties (Aditya and Bhattacharjee 2018), and the plants have incredible agronomic versatility (Rodriguez et al. 2020). The plants are considered autogamous but there are varying amounts of interspecific and inter-varietal hybridization (Suresh et al. 2014) that have further resulted in significant morphological and genetic diversity (Stetter et al. 2017) and a wider adaptability to different eco-geographical environments (Lee et al. 2008). Domestication syndrome remains indistinct in amaranth with strong photoperiod sensitivity and very small shattering seeds (Sauer 1967; Brenner et al. 2010). The frequent outcrossing with the wild-weedy relatives has not only complicated further the proper analysis of amaranth domestication (Stetter et al. 2016), but also led to a constraint for pure seed production (Brenner et al. 2013). The tiny intricate flowers make hand emasculation and pollination in amaranth difficult (Stetter et al. 2016) on a large scale. Alternative methods of hybridization include hot water treatment (Stetter et al. 2016) and the use of male sterility (Peters and Jain 1987; Brenner 2019). Although the application of hybrid breeding in amaranth is very promising (Lehmann et al. 1991), genetic improvement in the past has been achieved mainly through pedigree-based selection of suitable genotypes from landraces (Stetter et al. 2016). The major breeding objectives in amaranth include reduced seed shattering, reduced plant height (1.0–1.5 m), resistance to lodging, flowering above the leaf canopy for mechanical harvest, high grain yield, synchronized maturity, high grain quality, reduced leafiness in the green head area, and resistance to diseases and pests (Joshi et al. 2018).

The genetics of most of the agronomic traits have been determined (Joshi et al. 2018) including flowering time (Kulakow and Jain 1985), plant height, leaf length and width, panicle length and width (Kulakow and Jain 1987), harvest index, 1000 seed weight, grain yield (Pandey 1984), grain protein percentage (Pandey and Pal 1985), starch content of grain (Okuno and Sakoguchi 1982), seed coat color, inflorescence color and purple leaf mark (Gupta and Gudu 1990). Despite the availability of a 16-group SNP linkage map that has been constructed using an interspecific F_2_ mapping population between *A. hypochondriacus* x *A. caudatus* (Maughan et al. 2011), trait mapping has been limited due to the lack of robust mapping populations. The map comprises 411 SNP markers spanning 1288 cM with an average marker density of 3.1 cM per marker. The availability of a reference genome (Lightfoot et al. 2017) has made it possible to use SNP markers generated from genotyping-by-sequencing (GBS) for diversity analysis (Wu and Blair 2017) but is yet to be used for bi-parental or association mapping of traits.

### Grass pea

Grass pea (*Lathyrus sativus* L.) (2*n* = 2*x* = 14) is a member of the Fabaceae family. The genus consists of more than 150 species, which are further divided into 13 sections based on morphological traits (Kupicha 1983). Grass pea belongs to the section *Lathyrus* along with 33 other species and is the only cultivated pulse crop (Allkin 1986) in the genus. The primary gene pool consists of the highly variable *L. sativus* accessions (Yunus and Jackson 1991), while the secondary gene pool includes *L. amphicarpos, L. cicero, L. chrysanthus, L. gorgoni, L. marmoratus* and *L*. *pseudocicera*, *L. blepharicarpus*, *L. hierosolymitanus* and *L. hirsutus.* Grass pea cultivation originated around 6000 BC in the Balkan peninsula (Kislev 1989) and is believed to have been the first crop to be domesticated in Europe (Kislev 1989). Its production has now spread to other parts of the world, both temperate and tropical, including North and South America, the Canary Islands, the Mediterranean region, East Africa and Asia. Almost 20,000 accessions of *Lathyrus* spp. are maintained in different gene banks across 27 countries (Shehadeh 2011; Vaz Patto and Rubiales 2014), with a safe duplication of 3239 accessions in the global collection at the International Centre for Agricultural Research in Dryland Areas (ICARDA). The CROP TRUST (https://www.croptrust.org/crop/grass-pea/) is currently supporting 4,451 unique grass pea germplasm, of which 3595 are backed up at the Svalbard Global Seed Vault. France, India, Bangladesh and Chile hold the largest collections of *L. sativus* (Hillocks and Maruthi 2012) globally, while Ethiopia has the largest collection of grass pea in Africa (Girma and Korbu 2012).

Grass pea is cultivated for its seeds as a healthy food (Lambein et al. 2019) and feed (Smulikowska et al. 2008), as well as for fodder (Singh and Roy 2013). It is a hardy and resilient crop (Campbell 1997) that is rarely affected by pests and diseases (Rahman et al. 1995) and is one of the most climate and environmental change tolerant legumes (Kislev 1989; Yunus and Jackson 1991; Chowdhury and Slinkard 2000; Vaz Patto et al. 2006).

Grass pea is predominantly autogamous, although some level of cross-pollination has been reported (Rahman et al. 1995; Hanson and Street 2008; Ghorbel et al. 2014). Crop improvement has been achieved through conventional selection from landraces (Yunus and Jackson 1991; Vaz Patto et al. 2006), with the initial objective of improving domestication syndrome traits. Grass pea has also been included as a mandate crop of ICARDA, thereby enhancing its breeding structure. Finding high yielding cultivars with low ODAP content (< 0.2%) has been the main breeding objective (Campbell 1997; Girma and Korbu 2012) due to the high risk of lathyrism (Barone and Tulumello 2020) that would result from the consumption of high ODAP genotypes. High genetic variation for ODAP content, ranging from 0.02 to 2.59%, has been reported within and among populations of grass pea (Campbell 1997; Tay et al. 2000; Granati et al. 2003; Tadesse and Bekele 2003; Vaz Patto et al. 2006; Girma and Korbu 2012) that have led to the release of a number of cultivars with low ODAP (Campbell 1997; Granati et al. 2003; Tadesse and Bekele 2003; Vaz Patto et al. 2006; Girma and Korbu 2012; Hillocks and Maruthi 2012; Vaz Patto and Rubiales 2014) through conventional breeding. Hybridity is largely achieved through hand emasculation and pollination.

The first linkage map in grass pea was constructed using molecular and isozyme markers on an F_2_ population of 100 individuals derived from a cross between a blue-flowered and a white-flowered parent (Chowdhury and Slinkard 1999). The map comprised 71 RAPD, three isozymes and one morphological marker spread across 14 linkage groups and spanning 898 cM with an average distance between markers of 17.2 cM. About 12% of the markers used showed distorted segregation. The second linkage map was constructed using a backcross population of 92 individuals segregating for resistance to ascochyta blight (*Mycosphaerella pinodes*) (Skiba et al. 2004). The same study mapped two QTLs responsible for 12 and 9% of trait variation in linkage groups 1 and 2 using 47 RAPDs, 7 STMS and 13 STS/CAPS markers that spanned 803.1 cM across nine linkage groups. There are no records of any additional trait mapping studies either using bi-parental or diverse populations. Both simple sequence repeat (SSR) and SNP markers have been developed in grass pea (Yang et al. 2014; Hao et al. 2017), and a draft genome sequence is now available (Emmrich et al. 2020) to enable routine molecular analysis for trait mapping and characterization. More robust populations will also need to be developed to enable precise analysis of complex traits.

### Water yam

Water yam (*Dioscorea alata* L.) (2*n* = 2*x* = 40), also known as the greater yam, is one of the oldest cultivated yam species (Lebot 2009) and the most widely cultivated yam species worldwide (Abraham and Nair 1990; Obidiegwu et al. 2009). It belongs to the genus *Dioscorea* which comprises over 600 species distributed primarily in the tropics and subtropics (Rao et al. 1973)**.** The genus *Dioscorea* was historically assembled into 32–59 sections (Knuth 1924; Ayensu 1972). The section Enantiophyllum Uline is the most important section as it contains the three economically important species, *D. alata, D. cayenensis* and *D. rotundata*, all of which are cultivated worldwide. The other important species in the genus include *D. bulbifera, D. esculenta, D. opposita*, *D. japonica, D. nummularia, D. pentaphylla, D. transversa*, *D. trifida* and *D. dumetorum* (Dahiya et al. 2015; Efraín González Ramírez and García 2019). The species belonging to the Enantiophyllum section that includes water yam are considered unique to Southeast Asia (Malapa et al. 2005) suggesting they likely originated from this part of the world. *D. alata* is believed to have been domesticated about 6000 years ago (Lebot 2009) in Melanesia, where the greatest phenotypic variability has been observed (Lebot et al. 1998). Although previous studies reported that *D. alata* was close to *D. nummularia* and *D. transversa* (Malapa et al. 2005; Wilkin et al. 2005), this school of thought has been recently challenged (Caetano et al. 2016) and the true ancestry of *D. alata* remains unknown. Ex site germplasm collections have been assembled at the Central Tuber Crops Research Institute, Kerala, India (431 accessions), Centre de Ressources Biologiques Plantes Tropicales INRA-CIRAD, Guadeloupe, France (181) and at the International Institute of Tropical Agriculture (IITA) in Ibadan, Nigeria (772 accessions) (Arnau et al. 2017).

Water yam has a wide geographical distribution and is especially desirable for production due to high yield potential, ease of propagation, early growth vigor for weed suppression, long storability of tubers (Sartie and Asiedu 2014) and high nutritional content of tubers (Fauziah et al. 2020). It is dioecious (produces separate male and female plants) (Egesi et al. 2002; Obidiegwu et al. 2009; Ajayi and Oyetayo 2009; Baboli and Safe Kordi 2010) with ploidy levels ranging from diploids (2*n* = 2x = 40), triploids (2*n* = 3*x* = 60) and tetraploids (2*n* = 4*x* = 80) (Abraham and Gopinathan Nair 1991; Egesi et al. 2002; Obidiegwu et al. 2009; Arnau et al. 2009; Baboli and Safe Kordi 2010). Water yam is almost exclusively clonally propagated using small tubers or small pieces of tubers (Arnau et al. 2017), which provides agronomical advantages but has the disadvantage of enhancing the spread of diseases (Arnau et al. 2017). The major breeding objectives in water yam include resistance to anthracnose disease [*Colletotrichum gloeosporioides* (Penz.)] (Bhartiya et al. 2017), high tuber quality and yield potential (Arnau et al. 2016). Conventional hybridization through ploidy induction and manipulation (Arnau et al. 2010; Baboli and Safe Kordi 2010) has been successfully used to improve resistance to biotic and abiotic stresses (Darkwa et al. 2020), while higher vigor and tuber yield advantage were reported with tetraploid (2*n* = 4*x* = 80) and triploid (2*n* = 3*x* = 60) water yam compared to its diploid (2*n* = 2*x* = 40) counterpart (Arnau et al. 2007; Lebot 2009; Lebot et al. 2019). Artificially induced polyploidy has positive effects on chlorophyll content, leaf shape, stomata density, plant width, vine size and length and fruit (Kenji et al. 2005; Ajayi et al. 2010; Baboli and Safe Kordi 2010; Abraham et al. 2013). Successful interspecific hybridization that has been reported between *D. alata* and *D. nummularia* under artificial hand pollination (Lebot et al. 2017) provides further opportunities to introgress superior traits from *D. nummularia* such as resistance to anthracnose disease, high dry matter content of the tubers, high vigor and robustness, resistance to cyclones and tolerance to acid rain (Lebot et al. 2017). There are no reports of successful hybridization between *D. alata* and either *D. rotundata* and *D. cayenensis* (Rao et al. 1973; Arnau et al. 2007; Lopez-Montes et al. 2012).

Wide morphological and genetic variation has been reported in water yam (Arnau et al. 2017; Agre et al. 2019), which has been exploited in a few cases using molecular tools to identify genomic regions responsible for traits of interest. The first intraspecific genetic linkage map of *D*. *alata* was constructed using 523 polymorphic AFLP markers that were mapped onto 20 linkage groups spanning 1233 cM with a mean marker spacing of 2.31 cM (Mignouna et al. 2002). This linkage map also led to the identification of an AFLP marker linked to anthracnose resistance although only 10% of phenotypic variance was explained (Mignouna et al. 2002). Petro et al. (2011) later developed a more saturated AFLP linkage map and identified nine QTLs linked to anthracnose resistance that explained a range of 7–32.9% of phenotypic variance. More recently, Bhartiya et al. (2017) used EST-SSRs to map resistance to anthracnose and identified a consistent QTL on LG14 that explained 68.5% of the total phenotypic variation. The most recent linkage map of water yam was developed using SNP markers generated from genotyping-by-sequencing (GBS) and led to the identification of a major sex determination QTL on LG6 (Carrillo-Perdomo et al. 2019). Besides their use for linkage mapping, molecular markers have also been used in water yam for hybridity testing (Sartie and Asiedu 2011).

### African eggplant

The African eggplant (*Solanum aethiopicum*) (2*n* = 2*x* = 24) belongs to the Solanaceae family and genus *Solanum*. It is one of the only three cultivated eggplants together with the Gboma eggplant (*S*. *macrocarpon*) and brinjal eggplant (*S. melongena*)*,* all of which belong to the *Leptostemonum* clade, and to a species-rich sub-clade native to the Old World; Africa, Australia, and Asia (Lester and Daunay 2003; Acquadro et al. 2017). Studies based on seed protein and amplified fragment length polymorphism (AFLP) markers confirmed that *S. aethiopicum* is more related to *S*. *macrocarpon* than to *S. melongena* (Daunay et al. 2001; Sękara et al. 2007). The African eggplant is believed to have been domesticated in Africa from its wild progenitor *Solanum anguivi* (Sakata and Lester 1997; Lester 1998), which forms part of its primary genepool, and hybrids between *S. aethiopicum* and *S. anguivi* are fully fertile (Lester and Niakan 1986; Plazas et al. 2014; Taher et al. 2017). Successful crosses are possible between the African eggplant and both Gboma eggplant and brinjal eggplant, as well as with their respective ancestors *S*. *dasyphyllum* and *S*. *insanum* with intermediate fertility (Daunay et al. 1991; Prohens et al. 2012; Plazas et al. 2014). The three eggplants are also related to a large number of wild species (Syfert et al. 2016), which are well adapted to a wide range of conditions, from desert to swampy areas and environments with wide ranges of temperatures. GENESYS (2017) records 798 accessions of *S. aethiopicum* with possible additional collections in India and the Institute of Vegetables and Flowers, China (Taher et al. 2017). Although other unreported collections of *S. aethiopicum* may exist in different countries, more global collections need to be urgently done to avoid genetic erosion in this nutritious climate resilient vegetable crop. The African eggplant cultivation is mostly restricted to Africa, but is also cultivated in the Caribbean and Brazil (Schippers 2000) as well as in some areas of the southern part of Italy (Bukenya 1994; Sunseri et al. 2010).

The African eggplant is a hypervariate species (Lester et al. 1986; Plazas et al. 2014) with four recognized cultivar groups; Shum, Gilo, Kumba and Aculeatum (Lester 1986; Lester et al. 1986; Lester and Daunay 2003) that are completely inter-fertile (Lester and Niakan 1986). The Shum is used for its leaves; Kumba for both fruits and leaves; and Gilo for its fruits, and Aculeatum is used as an ornamental (Lester 1986; Schippers 2000; Lester and Daunay 2003). The crop is predominantly self-pollinating with up to 30% cross-pollination (Adeniji et al. 2012). It has been used as a source of resistance genes that have been introduced into other Solanaceae crops (Collonnier et al. 2001; Toppino et al. 2008; Rizza et al. 2002). Crop improvement has been achieved through the selection of landraces to enhance the domestication syndrome traits (non-shattering, reduced dormancy, increased seed size etc.), as well as improve resilience to biotic and abiotic stresses (Sseremba et al. 2018a, 2018b). There is now an established drought screening protocol (Nakanwagi et al. 2020) that can be used to identify drought resilient genotypes for use in generating relevant populations for future genetic studies. Its wild progenitor, *S. anguivi*, is a good source of novel alleles for disease resistance (Schippers 2000) and high number of fruits per inflorescence (Bukenya-Ziraba 2004; Osei et al. 2010; Afful et al. 2018).

The use of molecular markers within the African eggplant has been mainly for germplasm or genome characterization (Sakata et al. 1991; Sakata and Lester 1994; Gramazio et al. 2016; Song et al. 2019). There is currently no association or linkage mapping studies reported in the literature despite the recent availability of a reference genome (Song et al. 2019).

### Breadfruit

Breadfruit belongs to the genus *Artocarpus* (Moraceae), which consists of approximately 60 species native to the Oceania region (Kochummen 2000; Zerega et al. 2004). It is believed to have been domesticated from its wild ancestor, *Artilis camansi* Blanco (breadnut), in western Pacific about 3000 years ago (Ragone 2006; Zerega et al. 2006) from where it was spread by humans throughout the tropics (Roberts-Nkrumah 2007; Omubuwajo 2007; Ragone 1997). The cultivated breadfruit (*Artocarpus altilis,* (Parkinson) Fosberg, Moraceae), together with its wild relatives, *A. camansi*, *A. mariannensis* Trécul and natural hybrids (*A. altilis* x *A. mariannensis)* make up the breadfruit complex (Ragone 2007; Zerega et al. 2015). The National Tropical Botanical Garden (NTBG) in Hawaii is the main breadfruit conservation center and manages a field genebank of 220 accessions from 18 Pacific Island groups, the Philippines, the Seychelles, Indonesia and Honduras (Ragone 2007; Breadfruit Conservation Strategy 2007). Additional 33 accessions, including 24 duplicates from the NTBG collection, are maintained in the USDA/ARS National Plant Germplasm System at the Pacific Basin Tropical Plant Genetic Resources Management Unit in Hawaii and the National Germplasm Repository in Puerto Rico (Breadfruit Conservation Strategy 2007). There are other collections in Vanuatu (36 accessions), Samoa (200 collections from 14 countries), the University of the West Indies (33 accessions) (Ragone 2007) and several minor collections spread across the Pacific, Caribbean and West Africa (Breadfruit Conservation Strategy 2007). Morphological and molecular characterization of breadfruit collections and cultivars reveal a complex origin (Zerega et al. 2004) and high diversity (Sreekumar et al. 2007; Jones et al. 2012; Zerega et al. 2015).

Seedless cultivars of breadfruit, which are either triploids (2*n* = 3*x* =  ~ 84) or sterile diploids (2*n* = 2*x* = 56), are an important source of starch (Zerega et al. 2004) throughout Oceania, the Caribbean islands, and some parts of Africa and Asia. Breadfruit is grown mainly for its starchy fruit, which is a rich source of carbohydrates, fiber, vitamins, minerals flavonoids and complete protein (Rincon and Padilla 2004; Ijarotimi and Aroge 2005; Ragone and Cavaletto 2006; Jones et al. 2011, 2013; Liu et al. 2015). Different parts of the plant have pharmacological (Nwokocha et al. 2012; Jalal et al. 2015; Weng et al. 2018) and insect-repelling properties (Jones et al. 2012). Although seeded breadfruit cultivars can be propagated using seeds, vegetative propagation is the preferred method. Plants raised from seeds are not always true to type and lack uniformity (Ragone 2006; Deivanai and Bhore 2010). Vegetative propagation is done using rooted shootlets or root cuttings, air layering, budding and grafting onto seedling rootstocks (Deivanai and Bhore 2010). In vitro propagation using tissue culture has been optimized and is the preferred method of germplasm exchange besides its use for mass propagation (Murch et al. 2008). Breadfruit breeding objectives include improved resistance to lodging through wind damage during typhoons and cyclones (Daley et al. 2012; Zhou and Underhill 2019), resistance/tolerance to prolonged drought stress, resistance to mealybugs and breadfruit flies (*Bactrocera frauenfeldi* and *B. umbrosa*), fruit and root rots (*Phellinus noxius* and *Phytophthora palmivora)* (Ragone 2006; Zhou et al. 2014). Crop improvement has been achieved through traditional selection from landraces resulting in unique high yielding cultivars that can be distinguished morphologically (Lincoln and Ladefoged 2014). Yields of up to 50 tons/ha have been recorded (Roberts-Nkrumah 1998) despite the lack of agronomic and breeding research (Lincoln et al. 2018, 2019; Zhou et al. 2014).

Molecular studies in breadfruit have focused on understanding its evolution, domestication and overall genetic characterization using AFLPs (Sreekumar et al. 2007), RAPDs (Ifah et al. 2018), SSRs (De Bellis et al. 2016) and SNPs (Laricchia et al. 2018). Although a draft genome sequence (Sahu et al. 2019) was recently published, we found no record of association or linkage mapping studies. The availability of the draft genome provides a great opportunity for gene discovery, trait mapping and comparative genomics.

## Development of genetic and genomic resources in climate resilient orphan crops

Advances made in biotechnology and genomics, especially in next-generation sequencing (NGS), have significantly improved our understanding of orphan crops over the last two decades. Funded initiatives and web resources related to orphan crop genetics and genomics have become available (Padulosi 2017; Chiurugwi et al. 2019; Gregory et al. 2019; Jamnadass et al. 2020) leading to rapid genome characterization and candidate gene identification. There are now publicly available genome analysis tools enabling the utilization of resources from major crops for the exploitation of minor/orphan crops. There are also great opportunities to transfer the benefits of advanced breeding resources such as whole genome and transcriptome sequencing, genomic selection, genome editing and speed breeding from major crops to closely related climate resilient orphan crops. We discuss the processes and lessons to be learnt during the development of such resources in orphan crops under the following five main topics.

### Genomes and transcriptomes

Over the last five years alone, 30 orphan crops representing 13 families have had their genomes sequenced (Table 1). The selection criteria for genome sequencing included importance to local food security and nutritional value (Chang et al. 2019; Hendre et al. 2019; Jamnadass et al. 2020), and tolerance to environmental stresses (Song et al. 2019; Emmrich et al. 2020). The sizes of genomes sequenced ranged from 0.217 Gb (*Moringa oleifera*) to about 1.5 Gb (*Eleusine coracana*) (Table 1), which is relatively small compared to the full range of plant genome size (Liu et al. 2019). Only 8 out of the 30 genomes sequenced were polyploids (Table 1) highlighting a possible bias towards simple genomes, especially because the sequencing was mostly done using second-generation platforms, resulting in the assembly of draft genomes. Although some of these draft genomes will be more than adequate for utilization in molecular breeding, a third-generation (PacBio; Hi-C reads) sequencing tool will be needed to improve complex genomes as has been done for tef (VanBuren et al. 2020).

**Table 1:** Whole genome sequences of orphan crops generated since 2015

Family	Species	Ploidy	Estimated Genome size (Mbp)	N50 (kbp)	Reference
Amaranthaceae	*Amaranthus hypochondriacus*	2*X*	403	24,364	Lightfoot et al. (2017)
Anacardiaceae	*Sclerocarya birrea*	2*X*	331	335	Chang et al. (2018)
Dioscoreaceae	*Dioscorea alata*	2*X*	480	24,000	JGI (2020)
Dioscoreaceae	*Dioscorea dumetorum*	2*X*	322	3190	Siadjeu et al. (2020)
Dioscoreaceae	*Dioscorea rotundata*	2*X*	570	2120	Tamiru et al. (2017)
Fabaceae	*Vigna subterranean*	2*X*	535	641	Chang et al. (2018)
Fabaceae	*Lablab purpureus*	2*X*	395	621	Chang et al. (2018)
Fabaceae	*Faidherbia albida*	2*X*	654	692	Chang et al. (2018)
Fabaceae	*Lupinus angustifolius*	2*X*	609	13.8	Habiyaremye et al. (2017)
Fabaceae	*Vigna umbellata*	2*X*	415	207	Kaul et al. (2019)
Fabaceae	*Lupins albus*	2*X*	924	18,660	Xu et al. (2020), Hufnagel et al. (2020)
Fabaceae	*Vigna angularis*	2*X*	538	1290	Yang et al. (2014)
Fabaceae	*Vigna reflexo-pilosa*	4*X*	968	63	Yang et al. (2015)
Moraceae	*Artocarpus heterophyllus*	2*X*	982	548	Sahu et al. (2020)
Moraceae	*Artocarpus altilis*	2*X*	833	1536	Sahu et al. (2020)
Moringaceae	*Moringa oleifera*	2*X*	217	957	Chang et al. (2018)
Poaceae	*Digitaria exilis*	2*X*	716	10,741	Abrouk et al. (2020)
Poaceae	*Eragrostis tef*	4*X*	700	15,500	VanBuren et al. (2020)
Poaceae	*Eleusine coracana*	4*X*	1500	61,300	https://phytozome-next.jgi.doe.gov/info/Ecoracana_v1_1
Solanaceae	*Solanum aethiopicum*	2*X*	1020	516	Song et al. (2019)
Convolvulaceae	*Ipomoea batatas*	6*X*	873	6.5	Yan et al. (2015), Yang et al. (2017)
Poaceae	*Panicum miliaceum*	4*X*	923	369	Zou et al. (2019)
Polygonaceae	*Fagopyrum esculentum*	2*X*	1177	25.12	Yasui et al. (2016)
Amaranthaceae	*Chenopodium quinoa*	4*X*	1325	3846	Joseph et al. (2017)
Brassicaceae	*Brassica juncea*	4*X*	784	61	Yang et al. (2016), Pati et al. (2019)
Euphorbiaceae	*Jatropha curcas*	2*X*	339	145	Ha et al. (2019)
Cucurbitaceae	*Cucurbita maxima*	4*X*	271.4	3717	Sun et al. (2017)
Cucurbitaceae	*Momordica charantia*	2*X*	285.5	1100	Mayes et al. (2020), Urasaki et al. (2017)
Moraceae	*Morus alba*	2*X*	346.4	22,871	Luo et al. (2019), Jiao et al. (2020)
Cucurbitaceae	*Luffa cylindrica*	2*X*	416.3	53,000	Zhang et al. (2020)

Most whole genome sequencing projects are often coupled with the generation of the respective transcriptomes, which enable the full annotation of the genome generated. While the preferred transcriptome for annotation would be from the same species as was done for wild mustard (*Brassica juncea*) (Yang et al. 2016; Pati et al. 2019), the African eggplant (Song et al. 2019) and tef (VanBuren et al. 2020), there are also cases where the existing transcriptome of a close relative or a well-annotated transcriptome of a model crop has been used due to resource limitations. An earlier reference genome of finger millet, for example, was annotated using data from maize (Hittalmani et al. 2017). Other transcriptomes of orphan crops have been generated in response to specific biological questions, and the method of choice has been RNA sequencing (RNA-seq) (Ozsolak and Milos 2011). For example, Ranasinghe et al. (2019) identified 2416 differentially expressed genes while profiling for response to salt stress in quinoa (*Chenopodium quinoa*). In Jute-mallow, a transcription analysis was done to identify drought stress-related genes (Yang et al. 2017). Microarrays were the methods of choice for transcriptome analysis before the advent of NGS and were also applied in several orphan crops such as white lupin (Zhu et al. 2010), tef (Degu 2019), African nightshade (*Solanum nigrum*) (Schmidt and Baldwin 2009), wild mustard (Srivastava et al. 2015) and buckwheat (*Fagopyrum esculentum*) (Golisz et al. 2008) to detect expression profiles relevant to abiotic stress resilience.

### Molecular markers and genomic selection

A robust set of molecular markers is an important breeding resource in all crops but is often lacking in many orphan crops. Diversity Arrays Technology (DArT) (Jaccoud et al. 2001) has been one of the most relevant methods for molecular marker development in orphan crops as it is hybridization-based and therefore does not require prior sequence information. This technology transformed the genetic characterization and linkage mapping of a number of crops that were considered orphan two decades ago including pigeonpea (Yang et al. 2006; Yang et al. 2011) and cassava (*Manihot esculenta* Crantz) (Hurtado et al. 2008). More recently, DArT has been combined with NGS in a procedure called DArT-sequencing (DArT-seq) (Sansaloni et al. 2011) that enables high throughput genotyping for rapid SNP discovery in many orphan crops. DArT sequencing is now being used in the characterization of many climate-resilient orphan crops including Bambara groundnut (Redjeki et al. 2020), finger millet (Dida et al. 2020), Kersting’s groundnut (*Kerstingiella geocarpa*) (Kafoutchoni et al. 2020), lupin (*Lupinus albus*) (Raman et al. 2014) and grass pea (Almeida et al. 2016).

Aside from DArT-sequencing, other restriction-associated DNA sequencing (RADseq) (Davey et al. 2011) genotyping methods including genotyping-by-sequencing (GBS) (Elshire et al. 2011) have also been exploited in the characterization and linkage mapping of orphan crops, especially where a reference genome is available as was done in white Guinea yam (*Dioscorea rotundata*) (Tamiru et al. 2017). These sequence-based genotyping platforms are the future of genotyping in all crops including orphan crops with no reference genomes. With the dropping costs of NGS, most of the climate resilient orphan crops will most likely have their genomes sequenced in the next decade. Where whole genome sequences will not be available, comparative genomics (Feltus et al. 2006) could be exploited alongside other tools that would enable more precise SNP calling from NGS data in the absence of reference genomes (Lu et al. 2013; Russell et al. 2014; Melo et al. 2016).

Availability of a robust set of molecular markers would pave the way for genomic selection (GS), a form of marker-assisted selection that uses dense markers covering the whole genome to estimate the breeding value of selection candidates for a quantitative trait (Goddard 2009). Genomic selection promises to increase genetic gain in crops (Voss-Fels et al. 2019) and would therefore provide an opportunity for the much-needed progress in the crop improvement of orphan crops. A lot of successes have been reported in the implementation of GS in several crops including relatively underutilized crop species such as sorghum (Fernandes et al. 2018), cassava (Ozimati et al. 2018; Torres et al. 2019) and Kersting’s groundnut (Akohoue et al. 2020). For GS to work optimally in most of the climate resilient orphan crops, there will be need to develop data analysis tools that would enable the parallel analysis of NGS genotyping data alongside high-quality phenotypic data. Luckily, several digital tools and programs exist that support orphan crops including the Breeding Management System (BMS) (Shrestha et al. 2012), which is a product of the Integrated Breeding Platform (IBP; https://www.integratedbreeding.net). There are also training programs such as the African Plant Breeding Academy (http://pba.ucdavis.edu/PBA_in_Africa/) with the goal of training orphan crop breeders in the most advanced theory and technologies for plant breeding in support of critical decisions for crop improvement.

### Identification of climate smart genes in orphan crops for use in major crops

Several genes involved in the response to extreme stress conditions have been identified in orphan crops, and in some cases, used to improve major crops, or functionally validated in model crops such as *Arabidopsis thaliana* or *Nicotiana tabacum* (Table 2). Majority of the genes reported for drought and/or salt stress are transcription factors, which are known to play important roles in regulating response of plants to abiotic stresses (Joshi et al. 2016; van Zelm et al. 2020). *BjDREB1B*, a DREB gene cloned from *Brassica juncea*, led to the accumulation of higher levels of free proline in tobacco confirming its role in response to drought and salinity (Cong et al. 2008). Another DREB transcription factor, *VrDREB2A*, cloned from mung bean (*Vigna radiata*), significantly increased the tolerance of transgenic *Arabidopsis* plants to drought and salt stresses (Chen et al. 2016).

**Table 2:** Examples of climate smart genes identified from select orphan crops

Crop	Genes	Roles	Model/major crop	References
Pseudocereals				
Amaranth *(Amaranthus hypochondriacus)*	Seed albumin gene *AmA1*	Improve growth, production and protein content	Potatoes	Chakraborty et al. (2000)
	*A*ntimicrobial peptide gene *Ah-AMP*	Pathogen/disease resistance	Tobacco	Chen et al. (2003)
	Nuclear factor-Y NF-YC subunits gene *AhNFY-C*	Drought tolerance	Arabidopsis	Palmeros-Suárez et al. (2015)
	Group VII ethylene response factor transcription factor *AhERF*	Water-deficit tolerance	Arabidopsis	Massange-SaÂnchez et al. (2016)
Buckwheat (*Fagopyrum esculentum)*	*D*ehydration-responsive element *(*DREB) transcription factor*s FeDREB1*	Enhanced freezing and drought tolerance	Arabidopsis	Fang et al. (2015)
	Metallothionein type 3 FeMT3	Drought and oxidative stress defense gene		Samardˇzic et al. (2010)
	Basic helix-loop-helix *FtbHLH3*	Drought/oxidative stress		Yao et al. (2017)
	R2R3-MYB transcription factor gene *FtMYB9*	Drought and salt stresses		Gao et al. (2017)
	R2R3-MYB transcription factors gene *FtMYB13*	Drought/salt tolerance	Arabidopsis	Huang et al. (2018)
Chia (*Salvia hispanica L.*)	Fatty acid desaturase 2 genes *ShFAD2-1* and *ShFAD2-2*	Cold-induced and heat-repressed		Xue et al. (2017)
Quinoa *(Chenopodium quinoa)*	Salt Overly Sensitive 1 (SOS1) genes *cqSOS1A* and *cqSOS1B*	Salt tolerance		Maughan et al. (2009)
	Sodium transporter genes *CqSOS1* and *CqNHX*	Salt tolerance		Ruiz-Carrasco et al. (2011)
Vegetables				
Wild mustard *(Brassica juncea*)	Annexin protein *AnnBj1*	Tolerance to dehydration, salt, heavy metal and oxidative stress; pathogen resistance	Tobacco	Jami et al. (2008)
	Nonexpressor of pathogenesis-related genes 1 *BjNPR1*	Resistance against various pathogens	Rice	Sadumpati et al. (2013)
	*Y*ellow stripe-like gene *BjYSL7*	Increased heavy metal tolerance	Tobacco	Wang et al. (2013)
	Heat shock protein gene *HSP*	Drought stress		Aneja et al. (2015)
	*G*amma-glutamylcysteine synthetase genes *BrECS1* and *BrECS2*	Tolerance to abiotic stress and enhance growth and development	Rice	Bae et al. (2013)
Okra (*Abelmoschus esculentus)*	Chalcone synthasegene *AeCHS*		Arabidopsis	Wang et al. (2018)
Ethiopian kale *(Brassica carinata)*	DREB (dehydration responsive element binding protein) gene *BjDREB1B*	Drought, salt, low temperature, heavy metals		Cong et al. (2008)
Bitter gourd *(Momordica charantia)*	*C*lass I secretory endochitinase *McCHIT1*	Disease resistance	Rice	Li et al. (2009)
Bottle gourd *(Lagenaria siceraria)*	Gourd E3 ubiquitin ligase gene *LsRZF1*	Drought stress	Arabidopsis	Min et al. (2014)
Pumpkin (*Cucurbita maxima)*	Pumpkin phloem gene *CmPP16*	Response to drought stress		Ramírez-Ortega et al. (2014)
Wild melon (*Citrullus lanatus*)	NAM, ATAF1/2, and CUC2 (NAC) transcription factors gene *ClNAC*	Drought and salt stresses		Lv et al. (2016)
Sponge gourd (*Luffa cylindrical*)	*A*scorbate peroxidase *LcAPX*	Resistance to flooding	Arabidopsis	Chiang et al. (2017)
Legumes and pulses				
Broad/faba bean *(Vicia faba)*	Putative aquaporin gene *VfPIP1*	Drought tolerance	Arabidopsis	Cui et al. (2008)
Mung bean/ green Gram *(Vigna radiata)*	*P*hospholipase C gene *VrPLC*	Response to drought and salt tolerance		Gnanaraj et al. (2015)
	Dehydration-responsive element-binding protein 2 (DREB2) transcription factor gene *VrDREB2A*	Drought and salt stresses	Arabidopsis	Chen et al. (2016)
	*V*acuolar Na + /H + antiporter gen*e VrNHX1*	Salt tolerance	Cowpea	Mishra et al. (2014)
Horsegram (*Macrotyloma uniflorum*)	WRKY transcription factors gene *MuWRKY3*	Tolerance to drought stress	Groundnuts	Kiranmai et al. (2018)
	NAC transcription factor gene *MuNAC4*	Tolerance to drought stress	Groundnuts	Pandurangaiah et al. (2014)
	70-KD heat shock protein gene *MuHSP70*	Drought stress tolerant	Arabidopsis	Masand and Yadav (2016)
Hyacinth bean *(Lablab purpureus)*	A novel R2R3-MYB factor gene *LpMYB1*	Drought and salt tolerance	Arabidopsis	Yao et al. (2016)
Rice bean *(Vigna umbellata)*	C2H2-type zinc finger transcription factor gene *VuSTOP1*	pH and aluminum tolerance		Fan et al. (2015)
Oil Seeds				
Sesame *(Sesamum Indicum)*	Osmotin-like proteins gene *SindOLPs*	Tolerance to drought, salinity, oxidative stress, and the charcoal rot pathogen		Chowdhury et al. (2017)
Castor bean *(Ricinus communis)*	Vacuolar Na + /H + antiporter gene *SbNHX1*	Salt tolerance		Patel et al. (2015)
Cereals				
Finger millet *(Eleusine coracana)*	*B*asic helix-loop-helix transcription factors gene *EcbHLH*	Tolerance to salinity and drought stress	Tobacco	Babitha et al. (2015a)
	NAC proteins *EcNAC67*	Tolerance to salinity and drought stress	Rice	Rahman et al. (2016)
	*B*asic leucine zippers gene *EcbZIP60*	Tolerance to salinity and drought stress	Tobacco	Babitha et al. (2015b)
	G-BOX BINDING FACTOR 3 (GBF3) gene *EcGBF3*	Tolerance to osmotic stress, salinity and drought stress	Arabidopsis	Ramegowda et al. (2017)
	*E*ndoplasmic reticulum (ER) membrane tethered bZIP transcription factor gene *EcbZIP17*	Tolerance to various environmental stresses		
Foxtail millet (*Setaria italica*)	Abscisic acid (ABA)-responsive DREB-binding protein gene *SiARDP*	Drought tolerance		Li et al. (2014)
	Remorin gene *SiREM6*	Salt tolerance		Yue et al. (2014)
	Phospholipase D gene *SiPLDα1*	Drought tolerance	Arabidopsis	Peng et al. (2010)
Tubers				
Sweet potato (*Ipomoea batatas)*	Cysteine protease *SPCP2*	Senescence and extreme stress tolerance	Arabidopsis	Chen et al. (2010)
Water yam (*Dioscorea alata*)	Ascorbate peroxidase gene *DaAPX*	Tolerance to chilling, flooding, and oxidative stresses	Arabidopsis	Chen et al. (2019)
Fruits				
Bread fruit (*Artocarpus altilis*)	DELLA proteins genes *AaDELLA1* and *AaDELLA2*	Salinity tolerance		Zhou and Underhill (2017)
Physic nut *(Jatropha curcas*)	DREB transcription factor gene *JcDREB*	Salt and freezing stresses	Arabidopsis	Tang et al. (2011)
	Betaine aldehyde dehydrogenase gene *JcBD1*	Salt, drought and heat stresses		Zhang et al. (2008)

Management of pests and diseases has also benefitted from genes and other resources identified from orphan crops. Within the Solanaceae family, resistance to *Fusarium oxysporium* f. sp. *melongenae* (Rizza et al. 2002; Toppino et al. 2008) in brinjal eggplant was introduced from the African eggplant, while resistance to late blight (*Phytophthora infestans*) in potato (*Solanum tuberosum*) has been traditionally managed through the introgression of major genes from underutilized relatives (Ross 1986; Gebhardt and Valkonen 2001; Van Der Vossen et al. 2003; Ghislain et al. 2019). A recent whole genome analysis confirmed the abundance of disease resistance genes in the African eggplant (Song et al. 2019), making it a great resource for future introgression and *R* gene cloning. The African rice (*Oryza glaberrima* Steud.), an underutilized rice species cultivated in West Africa, is a major source of resistance to *Rice yellow mottle virus* (RYMV) (Pidon et al. 2017), bacterial blight (*Xanthomonas oryzae* pv. *Oryzae*) (Neelam et al. 2020), blast disease (*Magnaporthe oryzae)* (Dong et al. 2020), green rice leafhopper (*Nephotettix nigropictus*) (Fujita et al. 2010), as well as rice gall midge (*Orseolia oryzae*) (Ukwungwu et al. 1998) to the Asian rice.

An Ascorbate peroxidase (*APX*) gene cloned from yam (*Dioscorea alata*) was shown to enhance tolerance to flood and chilling stresses in transgenic *Arabidopsis* (Rosa et al. 2010; Bonifacio et al. 2011; Chen et al. 2019). A high-quality reference genome of mung bean enabled fast identification of synteny blocks associated with seed weight and nematode resistance through comparative analysis with soybean (*Glycine max*) and led to the development of functional markers for mung bean (Kang et al. 2014).

### Genome editing

Genome editing is a conventional method that is applied to alter the genotype and phenotype of organisms (Zhang et al. 2017) and involves the exploitation of both natural and induced mutations in crop improvement. Several genome editing tools are now available (Please see Hassanin et al. 2019) although the clustered regularly interspaced short palindromic repeats (CRISPR)–CRISPR-associated protein-9 nuclease (Cas9) (CRISPR–Cas9) (Doudna and Charpentier 2014) is the most common. Genome editing might be used to rapidly modify undesirable traits in orphan plants and accelerate the process of domestication. This can be through the reduction of the plant content of secondary metabolites, which are often toxic (Jørgensen et al. 2005; Østerberg et al. 2017). While genome editing using CRISPR-Cas9 has been suggested as a promising method for improving domestication syndrome traits in both diploids (Lemmon et al. 2018) and polyploids (Tripathi et al. 2019; Zaman et al. 2019), there are some limiting factors in orphan crops, including lack of a well-annotated genome, sub-optimal tissue culture regeneration protocols and lack of genetic transformation methods. For closely related crops, some of these techniques could be transferred from model and/or well-studied crops and replicated to achieve similar results in promising orphan crops in a fraction of the time that it took for the major crops (Chiurugwi et al. 2019; Pareek et al. 2020). Lemmon et al. (2009) successfully applied genome editing using CRISPR-Cas9 in a tomato wild relative, groundcherry (*Physalis pruinosa*) and improved domestication and productivity traits. An efficient CRISPR/Cas9‐based genome editing has also been established for banana (Kaur et al. 2017), a polyploid that is relatively under-researched, making it a good example for other orphan crops with complex genomes.

### Speed breeding

A recent review highlights speed breeding as one of the key technologies that would revolutionize the breeding of orphan crops (Chiurugwi et al. 2019). Proven methods of shortening the growth cycle of crops include the combination of at least two of the following; extending the duration of exposure to light (Ghosh et al. 2018), improved hand pollination and emasculation techniques (Stetter et al. 2016), growing the crops in a growth chamber (Ghosh et al. 2018), doubled haploidy (Chaudhary et al. 2019), optimal temperatures and humidity (Connor et al. 2013) and early seed harvest (Ghosh et al. 2018). Speed breeding protocols have been optimized for cereals such as wheat (*Triticum aestivum* L.) (Alahmad et al. 2018) and rice (*Oryza sativa* L.) (Ohnishi et al. 2011), and legumes including groundnut (*Arachis hypogaea* L.) (O'Connor et al. 2013) and chickpea (*Cicer arietinum* L.) (Samineni et al. 2020). These existing protocols could be tested in closely related orphan crops, perhaps with minor modifications. There is already a rapid production protocol for grain amaranths, which was developed through a combination of controlled growth conditions and efficient crossing methods (Stetter et al. 2016). Other protocols have been developed for grass pea and quinoa, which could soon lead to new varieties (Ghosh et al. 2018). Speed breeding, therefore, looks very promising and could be implemented right away to enhance orphan crops.

## Challenges in breeding climate smart orphan crops

### Lack of research investments

Despite the demonstrated importance of breeding orphan crops for climate change resilience and the progress that is being made, orphan crops still face several investment challenges. Most national, private sector and international agricultural research funding is skewed toward major crops (Naluwairo 2011). Rice, maize and wheat remain the highest priority crops in most countries (Shiferaw et al. 2013; Shrestha et al. 2019). The methodologies that are currently being used for priority setting for agricultural research investment rely on areas of production and numbers of beneficiaries, which often leave out climate smart orphan crops that may be the only source of livelihoods for some of the most vulnerable populations. A different framework for prioritizing agricultural research investment needs to be considered. There is increasing evidence of reductions in future productivity of major crops due to climate change (Lizumi and Ramankutty 2016; Zhao et al. 2017) that should be used to justify more investments in climate resilient orphan crops.

A better investment plan would enhance the development of genetic and genomic resources, improve crop breeding and enable the exploitation of the demonstrated benefits under different climate change scenarios. The first priority would be to train breeders in the use of advanced breeding tools, as is currently being done by the African Plant Breeding Academy. Functional networks of breeders working on same crops or addressing the same challenges would need to be formed to provide learning platforms that would reduce duplication of activities and enhance utilization of funds invested. The implementation of digital tools that would enhance the proper utilization and interpretation of both genotypic and phenotypic data will also need to be done before advanced breeding methods such as GS and speed breeding are implemented. The use of additional modern tools such as bioinformatics (Armstead et al. 2009), GS, mutational R gene enrichment sequencing (MutRenSeq; Steuernagel et al. 2016); genome editing (Lemmon et al. 2018), high throughput phenotyping (Mir et al. 2019) and nanotechnology (Jan et al. 2020) would make rapid improvement of orphan crops possible.

### Collection, documentation and characterization of germplasm

The rich diversity that exists in the majority of these orphan crops is threatened with extinction unless the germplasm is conserved and fully characterized (Bhattacharjee 2009). There have been some national and international efforts (Sogbohossou et al. 2018; Daley et al. 2020; http://www.ntbg.org/breadfruit/) to conserve a few orphan crops, but in most cases, the germplasm collections are not optimum and lacking full genetic characterization. An evaluation of the world's largest breadfruit germplasm collection found that approximately 50% of the typical East Polynesian seedless triploid cultivars were represented by a single genotype (Zerega et al. 2015). The genetic gains made from the Green Revolution (Evenson and Gollin 2003) have been attributed to the conservation, characterization and exchange of germplasm (Pingali 2012). Both in situ and ex situ germplasm collections will be required followed by full characterization to enable successful crop improvement.

### Underdeveloped extension services and seed systems

Agricultural extension services in most countries have been built around a few major crops and may be minimal or non-existent for orphan crops. Yet these services have been described as the main conduit for disseminating information on farm technologies, support rural adult learning and assist farmers in developing their farm technical and managerial skills (Danso-Abbeam et al. 2018). Extension agents also serve as feedback channels between farmers and the global research community with respect to proven best practices (Kabunga et al. 2011). In some cases, these services have been digitized and therefore capable of reaching farmers using mobile phones in some of the remotest of villages (Fu and Akter 2016), as long as the verified information on the target crop is available. There is need to structure extension services by region and target crops in order to provide the relevant information for orphan crops as well, especially those that are climate resilient and form a major part of livelihoods in specific regions. For example, teff is the most important cereal crop in Ethiopia (VanBuren et al. 2020), but still lacking adequate extension services (Teshome and Tegegne 2020) beyond the delivery of a package with improved variety and fertilizer (Abraham 2015).

Related to extension services are the seed systems. Orphan crops generally have underdeveloped seed systems (Mabhaudhi et al. 2019) that result in the recycling of poor quality seeds and subsequently, extremely low yields. For vegetatively propagated orphan crops, development of rapid regeneration protocols under sterile laboratory conditions may be necessary as has been done for breadfruit (Murch et al. 2008) to ensure the distribution of disease-free quality seedlings to farmers. This takes time and resources and would need the establishment of special laboratories and trained personnel. But even for sexually propagated orphan crops, different forms of quality seed supply should be tested and regulated to suit the needs of the crop and the targeted agro-ecologies (Ahmed et al. 2009).

### Marketing

The value chains for orphan crops are not well developed resulting in poor quality products that may be unattractive to the end-user and fetching way below the true value. Finger millet in East Africa, for example, can be processed and marketed as a high value malt drink or in the baking and breakfast cereals industry but is instead mainly marketed for household porridge preparation. Teff value chain is often described as untraceable (Amentae et al. 2016) and lacking in value addition (Lee 2018). The increasing demand for healthy products in the west and among the growing middle and upper class in developing nations has the potential to drive the demand for healthy orphan crops. However, value addition, better presentation and packaging (Opole 2019) will be needed for these products to appeal to consumers.

## Future perspectives

In order to reduce the impact of climate change, there is a need to shift away from global dependence on a limited number of crop species (Mayes et al. 2012). Several orphan crops continue to make a difference in the livelihoods of many households, especially with the increasing effects of climate change. Some crops such as cassava (ICGMC 2015), a perennial with extensive root systems, and chickpea (Garg et al. 2011; Jain et al. 2013), that were considered orphan crops two decades ago, are poised to become the new major crops under the future low inputs climate smart agriculture (CSA) regime. The next-generation crop plants need to be water and nutrient use efficient and have sustainable yields over a wider range of environmental conditions (Pareek et al. 2020). The potential that has been observed in several of these orphan crops will need to be translated for regular profitable production by an average farmer by improving their genetics and agronomy to meet the global demands. The wide range of tools and techniques to enhance sustainable crop production and resilience to climate change that have been developed for major crops will need to be tested and validated for use in orphan crops to fast track their performance. Conventional breeding alongside advanced tools such as GS, speed breeding and genome editing will play a big role in accelerating the process of domestication, through the reduction of toxic plant content (Jørgensen et al. 2005; Østerberg et al. 2017) and enhancing of phenotypes for better yields under climate smart agriculture (Chandrasekaran et al. 2016; Li et al. 2017; Lu and Jian-Kang 2017; Pareek et al. 2020). Value addition, better presentation and packaging of these crops and their products will go a long way in enhancing their adoption, especially with the increasing interest in healthy foods and the need to protect environments through the production of climate-smart crops. The success of future climate resilient crops will require a multidisciplinary research effort and multi-stakeholder funding prioritization.

## Supplementary Information

Below is the link to the electronic supplementary material.

Supplementary file1 (xls 45 kb)
