# Correction to: Genetics and breeding for climate change in Orphan crops

**DOI:** 10.1007/s00122-021-03904-0

**Published:** 2021-07-22

**Authors:** Sandra Ndagire Kamenya, Erick Owuor Mikwa, Bo Song, Damaris Achieng Odeny

**Affiliations:** 1 African Center of Excellence in Agroecology and Livelihood Systems, Uganda Martyrs University, Kampala, Uganda; 2 The International Crops Research Institute for the Semi-Arid Tropics – Eastern and Southern Africa, Nairobi, Kenya; 3 Shenzhen Branch, Guangdong Laboratory for Lingnan Modern Agriculture, Genome Analysis Laboratory of the Ministry of Agriculture, Agricultural Genomics Institute At Shenzhen, Chinese Academy of Agricultural Sciences, Shenzhen, 518060 People’s Republic of China

## Correction to: Theoretical and Applied Genetics (2021) 134:1787–1815 10.1007/s00122-020-03755-1

In the original publication, several references were given incorrectly, or missing in the reference list. The correct references are listed below.

Under the sub-heading “Genome Editing”, the definition of genome editing in the first sentence is linked to the wrong reference. The correct reference should be:

Zhang H, Zhang J, Lang Z et al. (2017) Genome editing—principles and applications for functional genomics research and crop improvement. Crit Rev Plant Sci 36:291–309. 10.1080/07352689.2017.1402989

All other corrections refer to Table 1:

Joseph et al. (2017) should be replaced with Jarvis et al. (2017). The full reference is:

Jarvis DE, Ho YS, Lightfoot DJ, Schmöckel SM et al. (2017) The genome of *Chenopodium quinoa*. Nature 542:307–312. 10.1038/nature213702

Yang et al. (2014) should be cited as Yang et al. (2015) and should be referenced as:

Yang K, Tian Z, Chen C, Luo L et al. (2015) Genome sequencing of adzuki bean (*Vigna angularis*) provides insight into high starch and low fat accumulation and domestication. Proc Natl Acad Sci U S A 112:13213–13218. 10.1073/pnas.142094911.

Yang et al. (2015) should be replaced with Kang et al. (2014). The full reference is:

Kang YJ, Kim SK, Kim MY, Lestari P et al. (2014) Genome sequence of mungbean and insights into evolution within *Vigna* species. Nat Comm 5(1):1–9.

Pati et al. (2019) should be replaced with Paritosh et al. (2019). The full reference is:

Paritosh K, Yadava SK, Singh P, Bhayana L et al. (2021) A chromosome-scale assembly of allotetraploid *Brassica juncea* (AABB) elucidates comparative architecture of the A and B genomes. Plant Biotechnol J 19:602–614. 10.1111/pbi.13492.

The authors wrongfully cited Mayes et al. (2020) in Table 1. This citation should be deleted.

The following references were omitted in the reference list despite appearing in Table 1:

Ha J, Shim S, Lee T, Kang YJ et al. (2019) Genome sequence of *Jatropha curcas* L., a non-edible biodiesel plant, provides a resource to improve seed-related traits. Plant Biotechnol J 17:517–530. 10.1111/pbi.12995.

Hufnagel B, Marques A, Soriano A, Marquès L et al. (2020) High-quality genome sequence of white lupin provides insight into soil exploration and seed quality. Nat Commun 11:1–12. 10.1038/s41467-019-14197-9.

Jiao F, Luo R, Dai X, Liu H et al. (2020) Chromosome-level reference genome and population genomic analysis provide insights into the evolution and improvement of domesticated mulberry (*Morus alba*). Mol Plant 13:1001–1012. 10.1016/j.molp.2020.05.005.

Kaul T, Eswaran M, Thangaraj A, Meyyazhagan A et al. (2019) Rice Bean (*Vigna umbellata*) draft genome sequence: unravelling the late flowering and unpalatability related genomic resources for efficient domestication of this underutilized crop. bioRxiv Prepr 1–41. 10.1101/816595.

Luo J, Wang Y, Zhao AZJ (2019) The complete chloroplast genome of *Morus alba* (Moraceae: Morus), the herbal medicine species in China. Mitochondrial DNA Part B Resour 4:2467–2468. 10.1080/23802359.2019.1638328.

Sun H, Wu S, Zhang G, Jiao C et al. (2017) Karyotype stability and unbiased fractionation in the paleo-allotetraploid cucurbita genomes. Mol Plant 10:1293–1306. 10.1016/j.molp.2017.09.003.

Urasaki N, Takagi H, Natsume S, Uemura A et al. (2017) Draft genome sequence of bitter gourd (*Momordica charantia*), a vegetable and medicinal plant in tropical and subtropical regions. DNA Res 24:51–58. 10.1093/dnares/dsw047.

Xu W, Zhang Q, Yuan W, Xu F et al. (2020) The genome evolution and low-phosphorus adaptation in white lupin. Nat Commun 11:1–13. 10.1038/s41467-020-14891-z.

Yan L, Lai X, Li X, Wei C, Tan X, Zhang Y (2015) Analyses of the complete genome and gene expression of chloroplast of sweet potato [Ipomoea batata]. PLoS One 10:1–25. 10.1371/journal.pone.0124083.

Yang J, Liu D, Wang X, Ji C et al. (2016) The genome sequence of allopolyploid Brassica juncea and analysis of differential homoeolog gene expression influencing selection. Nat Genet 48:1225–1232. 10.1038/ng.3657.

Yang J, Moeinzadeh MH, Kuhl H, Helmuth J et al. (2017) Haplotype-resolved sweet potato genome traces back its hexaploidization history. Nat Plants 3:696–703. 10.1038/s41477-017-0002-z.

Yasui Y, Hirakawa H, Ueno M, Matsui K et al. (2016) Assembly of the draft genome of buckwheat and its applications in identifying agronomically useful genes. DNA Res 23:215–224. 10.1093/dnares/dsw012.

Zhang T, Ren X, Zhang Z, Ming Y et al. (2020) Long-read sequencing and de novo assembly of the *Luffa cylindrica* (L.) Roem. genome. Mol Ecol Resour 20:511–519. 10.1111/1755-0998.13129.

Zou C, Li L, Miki D, Li D et al. (2019) The genome of broomcorn millet. Nat Commun 10:1–11. 10.1038/s41467-019-08409-5.

